# Evaluation of bone density and skeletal muscle mass after sleeve gastrectomy using computed tomography method

**DOI:** 10.1016/j.bonr.2023.101661

**Published:** 2023-02-11

**Authors:** Kazuhiro Kai, Toshifumi Fujiwara, Yoshihiro Nagao, Eiji Oki, Tomoharu Yoshizumi, Masatoshi Eto, Yasuharu Nakashima

**Affiliations:** a Department of Orthopedic Surgery, Graduate School of Medical Sciences, Kyushu University, Fukuoka, Japan; b Department of Surgery and Science, Graduate School of Medical Science, Kyushu University, Fukuoka, Japan; c Department of Advanced Medical Initiatives, Graduate School of Medical Science, Kyushu University, Fukuoka, Japan

**Keywords:** CT, computed tomography, DXA, dual-energy X-ray absorptiometry, BMD, bone mineral density, BMI, body mass index, HbA1c, hemoglobin A1c, HU, Hounsfield unit, PMI, psoas muscle mass index, BIA, bioelectrical impedance analysis, Obesity, Sleeve gastrectomy, Bone mineral density (BMD), Computed tomography (CT), Hounsfield unit (HU), Psoas muscle mass index (PMI)

## Abstract

**Introduction:**

Sleeve gastrectomy is the most common surgical procedure to reduce weight and treat metabolic complications in patients with moderate-to-severe obesity; however, it affects the musculoskeletal system. Dual-energy X-ray absorptiometry (DXA), which is commonly used to measure bone mineral density (BMD), may be affected by excess fat tissue around the bones, interrupting BMD measurement. Due to the strong correlation between DXA and the Hounsfield units (HU) obtained from computed tomography (CT) scans, BMD assessment using clinical abdominal CT scans has been useful. To date, there has been no report of detailed CT evaluation in patients with severe obesity after sleeve gastrectomy.

**Objective:**

This study investigated the effect of sleeve gastrectomy in severely obese patients on bone and psoas muscle density, and cross-sectional area using retrospective clinical CT scans.

**Methods:**

This was a retrospective observational study that included 86 patients (35 males and 51 females) who underwent sleeve gastrectomy between March 2012 and May 2019. Patients' clinical data (age at the time of surgery, sex, body weight, body mass index (BMI), comorbidities, and preoperative and postoperative blood test results, HU of the lumbar spine and psoas muscle and psoas muscle mass index (PMI)) were evaluated.

**Results:**

The mean age at the time of surgery was 43 years, and the body weight and BMI significantly reduced (*p* < 0.01) after surgery. The mean hemoglobin A1c level showed significant improvement in males and females. Serum calcium and phosphorus levels remained unchanged before and after surgery. In CT analysis, HU of the lumbar spine and psoas muscle showed no significant decrease, but PMI showed a significant decrease (*p* < 0.01).

**Conclusions:**

Sleeve gastrectomy could dramatically improve anthropometric measures without causing changes in serum calcium and phosphorus levels. Preoperative and postoperative abdominal CT revealed no significant difference in the bone and psoas muscle density, and the psoas muscle mass was significantly decreased after sleeve gastrectomy.

## Highlights

• Perioperative changes of bone and muscle mass were assessed by computed tomography.
• Sleeve gastrectomy improves body weight and hemoglobinA1c.
• Sleeve gastrectomy affected muscle mass but not bone volume.

## 1 Introduction

Due to the increase of the obese population in Japan, metabolic and bariatric surgery, such as sleeve gastrectomy and Roux-en-Y gastric bypass surgery, has become an increasingly effective strategy for patients with moderate to severe obesity. Sleeve gastrectomy involves vertical resection of a major part of the stomach, and a tubular remnant is retained along the lesser curvature. In Roux-en-Y gastric bypass surgery, a small gastric pouch is connected to the small intestine, creating a bypass of the stomach, duodenum, and proximal jejunum (Peterli et al., 2018). Bariatric surgery affects bone metabolism, and Roux-en-Y gastric bypass surgery can possibly decrease bone density to a greater extent than sleeve gastrectomy (Jaruvongvanich et al., 2019; Bredella et al., 2017). Roux-en-Y gastric bypass surgery may result in malabsorption, leading to vitamin D deficiency and secondary hyperparathyroidism, whereas sleeve gastrectomy exerts a less effect on bone metabolism, such as serum calcium and serum 25-hydroxyvitamin D (Jaruvongvanich et al., 2019). However, some studies (Bredella et al., 2017; Santos et al., 2019) have used postoperative supplementation of calcium or vitamin D, whereas some have not (Damms-Machado et al., 2012; Lancha et al., 2014). Therefore, the effect of sleeve gastrectomy on bone metabolism has still remains unclear.

Sleeve gastrectomy can affect bone mineral density (BMD). Some studies showed that sleeve gastrectomy decreased the BMD of the lumbar spine, total proximal femur, and femoral neck (Jaruvongvanich et al., 2019; Bredella et al., 2017; Gagnon and Schafer, 2018; Hofso et al., 2021), as well as resulted in the loss of muscle mass (Kenngott et al., 2019); conversely, other studies reported no changed in BMD of the lumbar spine after sleeve gastrectomy (Ruiz-Tovar et al., 2013). Therefore, there is still a lack of consensus on the postoperative impact of sleeve gastrectomy on bones. BMD correlates with fracture risk (Marshall et al., 1996), and dual-energy X-ray absorptiometry (DXA) is the most commonly used method to determine BMD in patients with severe obesity after sleeve gastrectomy (Jaruvongvanich et al., 2019; Bredella et al., 2017; Gagnon and Schafer, 2018; Hofso et al., 2021). However, because DXA is susceptible to artifactual changes following extreme weight loss by sleeve gastrectomy, it remains unclear whether it is an accurate assessment method (Schreiber et al., 2011). Computed tomography (CT) scans automatically adjust the exposure time according to the patient's body size. This technology results in a more homogeneous energy spectrum of the X-ray beam encountered by the spine and enables an accurate measurement of the Hounsfield units (HU) of the target tissue (Schreiber et al., 2011). Due to the strong correlation between DXA and HU obtained from clinical abdominal CT scans, studies have reported that BMD assessment using clinical CT scans is useful (Schreiber et al., 2011; Lee et al., 2013; Lucas et al., 2017). To date, there has been no report of detailed CT evaluations in patients with severe obesity who underwent sleeve gastrectomy.

The aim of this study was to investigate the effects of sleeve gastrectomy on bone and psoas muscle density and psoas muscle cross-sectional area using retrospective clinical CT scans in patients with severe obesity.

## 2 Materials and methods

### 2.1 Study participants

Using a retrospective study design, we reviewed the medical records of all consecutive patients who underwent sleeve gastrectomy at Kyushu University Hospital between March 1, 2012, and May 31, 2019. Patients' clinical data (age at the time of surgery, sex, body weight, body mass index (BMI), comorbidities, and preoperative and postoperative blood test results (i.e., hemoglobin A1c (HbA1c), calcium, and phosphorus levels)) were extracted. We analyzed the preoperative and postoperative abdominal CT imaging results of patients. This retrospective study was approved by the Ethics Committee of our hospital (approval number: 2109-183).

### 2.2 Nutritional and exercise therapy

All patients underwent daily caloric intake restrictions of up to 300 kcal for the first postoperative month, 600 kcal for the next 2 months, 900 kcal for the next 4 months, and 1200 kcal thereafter. Calcium and vitamin supplements were not regularly prescribed, and supplement intake was recommended in the case of decreased serum calcium levels. Individualized postoperative rehabilitation was provided by our physical therapists during hospitalization, including routine experiential strength training and gait training.

### 2.3 Patients included in the analysis

Between March 2012 and May 2019, 164 patients with severe obesity underwent metabolic and bariatric surgery, which was performed according to the National Institutes of Health (NIH) criteria (i.e., BMI > 40 or >35 kg/m^2^ with comorbidities) (Mechanick et al., 2009). Postoperative CT examination at 1 year was performed for 93 patients. We included patients who underwent sleeve gastrectomy and excluded eight patients who underwent Roux-en-Y gastric surgery. A total of 86 patients underwent both preoperative and postoperative abdominal CT scans. No patients took medication for osteoporosis, and no patients became pregnant during observation.

### 2.4 CT analysis

**Fig. 1:**
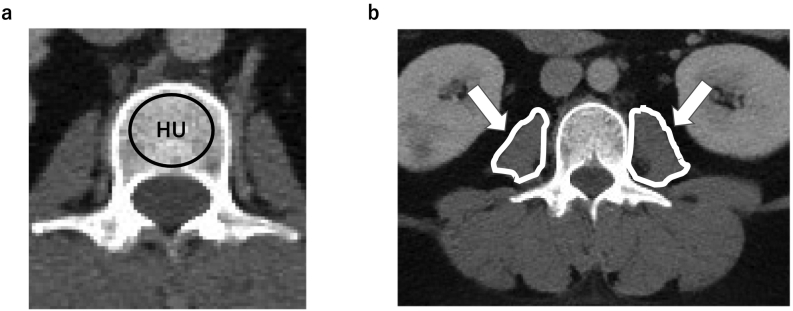
(a) Computed tomography (CT) scans illustrating the method of determining the HU value using the elliptical region of interest function. The image shows the axial plane of the middle of the body of L2. The HU values are generated using the imaging software program. (b) The cross-sectional areas of the right and left psoas muscles are measured by manual tracing from preoperative CT images in the middle of the body of L2, L3, and L4. The right and left psoas muscles are indicated by the white arrow. The psoas muscle mass index is calculated by normalizing the cross-sectional areas for height (cm^2^/m^2^).

For BMD assessment, we used HU (Schreiber et al., 2011), which was obtained from both preoperative and postoperative abdominal CT scans for clinical indications. For quantitative CT, helical sixty-four-channel CT scanners (Aquilion One; TOSHIBA, IQon Spectral CT; Philips) were used for all patients. The CT parameters included a slice thickness of 5 mm with a 0.625-mm interval, a tube voltage of 120 kVp, and a tube current of 350 mA. Quantitative CT was performed without the use of phantoms. The average value of HU was calculated by placing an elliptical region of interest that was confined to the medullary space of the vertebral body. Regions of interest were measured on the axial images at the middle of the vertebral body of L2 through L4 without cortical margins (Fig. 1). Only one slice per the vertebral body (L2, L3, L4) was analyzed simply. The values of HU from the three axial slices were averaged. All patients underwent preoperative CT scans, and postoperative CT was performed at an average of 1.2 ± 0.6 (range: 0.3–3.4) years following surgery. To quantify the skeletal muscle mass, the psoas muscle mass index (PMI) was used, as described by Hamaguchi et al. The cross-sectional areas of the right and left psoas muscles were measured by manual tracing at the L3 level of preoperative and postoperative CT images; subsequently, PMI was calculated by normalizing the cross-sectional areas for height (cm^2^/m^2^) (Hamaguchi et al., 2014). Furthermore, the HU of the psoas muscle was measured to determine the quality of muscle by manual tracing at the L3 level of preoperative and postoperative CT images.

### 2.5 Statistical analysis

Continuous and categorical variables were expressed as mean, range, median, and percentage. To compare with preoperative and postoperative variables, continuous variables were analyzed by the paired *t*-test. In male or female, continuous variables were analyzed by the Mann–Whitney *U* test. All statistical analyses were conducted using the JMP software (version 14.0.0, SAS Institute Inc., Cary, NC, USA), and a *p* value of <0.05 was considered statistically significant. Data are expressed as mean ± standard deviation (SD).

## 3 Results

### 3.1 Baseline characteristics

We analyzed the data of 86 consecutive patients who underwent sleeve gastrectomy (Table 1). The mean age at the time of surgery was 46.3 ± 10.3 (range: 18–76) years. Of the 86 patients, there were 35 (41 %) males, and 51 (59 %) females, and their mean BMI values were 44.1 ± 6.1 (range: 34.8–59.3) and 41.6 ± 6.1 (range: 35.1–61.9) kg/m^2^, respectively. Regarding comorbidities, diabetes mellitus, hypertension, and hyperlipidemia were observed in 73 (85 %) patients (32 males, 41 females), 64 (74 %) patients (26 males, 38 females), and 66 (77 %) patients (25 males, 41 females), respectively.

**Table 1:** Patient's baseline characteristics at the operation.

	Total N = 86	Male N = 35	Female N = 51	*p*-Value (male vs female)
Age (years)	46.3 ± 10.3 (18–76)	43.8 ± 10.2 (18–76)	48.1 ± 10.0 (21–73)	0.05
Weight (kg)	114.8 ± 23.8 (70–181.8)	131.3 ± 21.5 (93–181.8)	102.4 ± 16.7 (70–135.6)	0.12
Body mass index (kg/m^2^)	42.6 ± 6.2 (34.8–59.3)	44.1 ± 6.1 (34.8–59.3)	41.6 ± 6.1 (35.1–61.9)	0.17
Type 2 diabetes	73 (84.8)	32 (91.4)	41 (80)	0.13
Hyperextension	64 (74.4)	26 (74.2)	38 (74.5)	0.98
Hyperlipidemia	66 (76.7)	25 (71.4)	41 (80)	0.35
Hemoglobin A1c (%)	7.0 ± 1.4 (5.2–12.2)	7.1 ± 1.1 (5.3–9.3)	7.0 ± 1.6 (5.2–12.2)	0.86
Serum calcium (mg/dL)	9.3 ± 0.3 (8.6–10.1)	9.3 ± 0.3 (8.8–10.1)	9.3 ± 0.34 (8.6–10.1)	0.97
Serum phosphorus (mg/dL)	3.5 ± 0.5 (2.4–4.5)	3.4 ± 0.7 (2.4–4.3)	3.6 ± 0.4 (2.9–4.5)	0.43
HU in the vertebral body	177 ± 46.1 (74–266.3)	180 ± 44.3 (115.3–258.3)	176 ± 47.3 (74–266.3)	0.68
HU in the psoas muscle	56.4 ± 14.5 (21.3–93.5)	59.5 ± 16.3 (29.8–93.5)	54.2 ± 12.7 (21.3–90.8)	0.12
Psoas muscle index	5.7 ± 2.2 (2.0–12.3)	7.4 ± 2.0 (4.4–12.3)	4.6 ± 1.4 (2.0–8.6)	<0.01

Note: Results are shown as the mean ± standard deviation of the mean (range) or n (%).

Mann-Whitney analysis. *p* < 0.05 was statistically significant.

HU: Hounsfield unit.

### 3.2 Postoperative effects of bariatric surgery on anthropometric measures and body composition

Table 2 shows a comparison of preoperative and postoperative (after 1 year) anthropometric measures and body composition. The mean body weight was significantly decreased in both males (131.3 vs. 98.1 kg, *p* < 0.01) and females (102.4 vs. 74.0 kg, *p* < 0.01); furthermore, the mean BMI was significantly decreased in both males (43.6 vs. 33.0 kg/m^2^, *p* < 0.01) and female (41.6 vs. 30.1 kg/m^2^, *p* < 0.01). Studies have reported that bariatric surgery for the obese patients with diabetes was more effective than intensive medical therapy alone (Schauer et al., 2014), and reduced HbA1c levels (Switzer et al., 2016). Among patients with diabetes, higher fat content was associated with higher HbA1c levels, and body composition exerts a favorable effect on HbA1c levels (Bower et al., 2017). Therefore, we measured perioperative HbA1c levels to evaluate the body composition and observed that the mean HbA1c level significantly improved in both males (7.1 vs. 5.7, *p* < 0.01) and females (7.0 vs. 5.8, *p* < 0.01). Serum calcium and phosphorus levels remained unchanged preoperatively and postoperatively.

**Table 2:** Comparison of preoperative and postoperative body composition and hormones.

	Male			Female		
	Preoperative	Postoperative	*p*-Value	Preoperative	Postoperative	*p*-Value
Weight (kg)	131.3 ± 21.5 (93–181.8)	98.1 ± 16.0 (76.1–151.4)	<0.01	102.4 ± 16.7 (70–135.6)	74.0 ± 16.5 (44–120.4)	<0.01
Body mass index (kg/m^2^)	43.6 ± 6.4 (34.8–59.3)	33.0 ± 4.8 (25.4–43.1)	<0.01	41.6 ± 6.1 (35.1–61.9)	30.1 ± 6.1 (19.8–50.2)	<0.01
Hemoglobin A1c (%)	7.1 ± 1.1 (5.3–9.3)	5.7 ± 0.6 (4.8–7.7)	<0.01	7.0 ± 1.6 (5.2–12.2)	5.8 ± 0.7 (4.6–8.5)	<0.01
Serum calcium (mg/dL)	9.3 ± 0.3 (8.8–10.1)	9.3 ± 0.3 (8.1–10.1)	0.36	9.3 ± 0.3 (8.6–10.1)	9.2 ± 0.3 (8.6–10.2)	0.13
Serum phosphorus (mg/dL)	3.4 ± 0.7 (2.4–4.3)	3.4 ± 1.0 (2.5–4.2)	0.92	3.6 ± 0.4 (2.9–4.5)	3.5 ± 0.4 (1.7–4.4)	0.67
HU in the vertebral body	180 ± 44.3 (115.3–295.6)	172 ± 36.3 (115.3–258.3)	0.43	176 ± 47.3 (74–266.3)	166 ± 50.4 (61.3–297.3)	0.33
HU in the psoas muscle	59.5 ± 16.3 (29.8–93.5)	54 ± 7.2 (31.1–68.9)	0.07	54.2 ± 12.7 (21.3–90.8)	52 ± 9.6 (14.2–72.1)	0.32
Psoas muscle index	7.4 ± 2.0 (4.4–12.3)	6.4 ± 1.7 (2.1–11.0)	<0.01	4.6 ± 1.4 (2–8.3)	3.7 ± 1.4 (1.4–7.7)	<0.01

Note: Results are shown as the mean ± standard deviation of the mean (range).

Paired *t-*test (preoperative vs postoperative). *p* < 0.05 was statistically significant.

HU: Hounsfield unit.

### 3.3 Postoperative effects of bariatric surgery on the bone and muscle using CT image

Regarding BMD analysis, there was no significantly difference in the means of preoperative and postoperative HU values in the vertebral body of L2 through L4 in both of male and female patients (males: 180 ± 44.3–172 ± 36.3, *p* = 0.43; females: 176 ± 47.3–166 ± 50.4, *p* = 0.33). However, in the muscle mass analysis, the average of PMI for males significantly decreased from 7.4 ± 2.0 (range: 4.4–12.3) to 6.4 ± 1.7 (range: 2.1–11.0) cm^2^/m^2^ (*p* < 0.01). For females, the average of PMI was also significantly decreased from 4.6 ± 1.4 (range: 2–8.3) to 3.7 ± 1.4 (range: 1.4–7.7) cm^2^/m^2^ (*p* < 0.01) (Table 2). In the assessment of psoas muscle quality, the means of preoperative and postoperative HU values of the psoas muscle at the L3 level in male and female patients showed no significant difference, respectively (males: 59.5 ± 16.3 to 54 ± 7.2, *p* = 0.07; females: 54.2 ± 12.7 to 52 ± 9.6, *p* = 0.32).

## 4 Discussion

This retrospective study analyzed the changes in the density of the lumbar spine and psoas muscle, and the psoas muscle mass among adults with moderate-to-severe obesity who underwent sleeve gastrectomy for bariatric surgery using CT images. We found that weight reduction by the sleeve gastrectomy exerted no effect on the bone density of the lumbar spine and psoas muscle density but decreased the psoas muscle mass 1 year postoperatively.

With the increasing number of adults with moderate-to-severe obesity (Ezzati, 2016), bariatric surgery, such as sleeve gastrectomy, has become the most commonly performed procedure due to the advantages of weight loss, glycemic control, energy metabolism, and nutrition (Schauer et al., 2014; Griggs et al., 2018; Misra et al., 2020). Our study showed that body weight, BMI, and HbA1c levels significantly improved after sleeve gastrectomy. Previous studies reported that patients who underwent sleeve gastrectomy had maintained their serum calcium and phosphorus levels with strict vitamin D supplementation (Jaruvongvanich et al., 2019; Misra et al., 2020) and that bariatric surgery was associated with hyperparathyroidism, vitamin D deficiency, and lower calcium levels (Altawil et al., 2021), indicating the need to perform routine testing for parathyroid hormone and vitamin D. Our study demonstrated no significant changes in serum calcium and phosphorus levels without calcium or vitamin D supplementation.

As previously reported, body weight and bone volume show a positive correlation, and weight loss may affect bone volume (Slemenda et al., 1990; Compston et al., 1992; Lindsay et al., 1992). In Particular, a significant decrease in postoperative BMD as assessed by DXA in the lumbar spine, total proximal femur, and femoral neck was observed after sleeve gastrectomy due to the reduction of mechanical load on the skeleton owing to weight loss (Bredella et al., 2017; Liu et al., 2016). Conversely, some studies reported no loss in BMD as assessed by DXA after sleeve gastrectomy in the lumbar spine (Jaruvongvanich et al., 2019; Ben-Porat et al., 2022), suggesting that the effect on bones after sleeve gastrectomy remains controversial. Most studies examining BMD of the lumbar spine, total proximal femur, and femoral neck after sleeve gastrectomy have used DXA (Jaruvongvanich et al., 2019; Bredella et al., 2017; Ruiz-Tovar et al., 2013), which may fail to obtain accurate measurements in patients with severe obesity because the excess fat tissue around the bones could interrupt the measurement (Tothill et al., 1997; Bolotin, 1998). A previous study on the analysis of postoperative 2-year BMD by DXA after sleeve gastrectomy reported lower postoperative BMD of the total proximal femur and femoral neck, but not in the lumbar spine (Ben-Porat et al., 2022). In this study, we analyzed the BMD of the lumbar spine using preoperative and postoperative abdominal CT scans.

Previous evidence demonstrates that CT has been a useful alternative method to measure BMD using HU (Schreiber et al., 2011). In particular, CT could reveal bone and muscle density and muscle cross-sectional area due to less susceptible changes in body size, making it a possible useful method for patients with obesity (Yu et al., 2012). In this study, preoperative and postoperative examinations of abdominal CT scans were performed as routine tests for preoperative planning and postoperative evaluation and to obtain bone and psoas muscle density and psoas muscle cross-sectional area with relative ease. Our study, using HU obtained from abdominal CT scans, showed the same bone density of the lumbar spine after sleeve gastrectomy despite weight loss, which is consistent with that reported in previous studies (Jaruvongvanich et al., 2019; Ben-Porat et al., 2022). Roux-en-Y gastric bypass surgery resulted in lower postoperative BMD as assessed by DXA in the total proximal femur, femoral neck, and lumbar spine and higher fracture risk than sleeve gastrectomy (Hofso et al., 2021; Cadart et al., 2020). Because sleeve gastrectomy decreases gastric volume, food digestion and absorption may be unaffected, showing that our BMD results obtained using the HU of the lumbar spine also remained unchanged. Our study revealed no significant changes in the BMD of the lumbar spine evaluated by CT imaging, which is consistent with the results of previous studies that reported no significant difference preoperatively and postoperatively in the BMD of the lumbar spine after sleeve gastrectomy (Jaruvongvanich et al., 2019; Ben-Porat et al., 2022).

Muscle mass and body composition are associated with BMD (Moize et al., 2013; Vaurs et al., 2015); however, the detailed underlying mechanism remains unknown. There are several methods to evaluate skeletal muscle mass, including bioelectrical impedance analysis (BIA), CT, and DXA. BIA, which has been used to evaluate muscle mass in several studies (Ozeki et al., 2018), may be inaccurate in patients with obesity as the excess fat tissue surrounding the bone and water content could result in measurement errors. In this study, we evaluated patients who underwent sleeve gastrectomy using abdominal CT images. The PMI evaluation method is a CT-based muscle mass evaluation method, which was considered to be capable of pseudo-measuring whole-body skeletal muscle mass (Hamaguchi et al., 2014) and evaluating sarcopenia in the surgical field. Due to its correlation with BIA, PMI has been established as a useful and accurate muscle mass assessment method (Hamaguchi et al., 2014; Kobayashi et al., 2016). In our study, we observed a significantly decreased psoas muscle mass 1 year after sleeve gastrectomy. Although several studies have demonstrated decreased muscle mass after surgery (Vaurs et al., 2015) (Kobayashi et al., 2016; Stegen et al., 2011), functional evaluation was not influenced despite the reduction in skeletal muscle mass after surgery (Coral et al., 2021). Regarding peripheral muscles, muscle strength, including grip strength, decreases after sleeve surgery and RYGB (Otto et al., 2014). In this study, the muscle cross-sectional area of the psoas major muscle measured by CT was found to be reduced. The psoas major muscle flexes the hip joint and spinal column and is important for gait and maintaining posture. Therefore, the impairment of the psoas muscle may lead to gait disorder (Evans, 2010). In our study, although we did not examine the functional outcome such as psoas major and exercise, no patients complained of poor muscle mass, such as fatigue and muscle weakness.

Sleeve gastrectomy and Roux-en-Y gastric bypass surgery are known to influence skeletal muscle mass (Levitt et al., 2010; Moize et al., 2013), and low skeletal muscle mass has been observed in >25 % of cases after surgery, resulting in an association with the risk of developing sarcopenia (Molero et al., 2022). In contrast, although adults with obesity have higher muscle volume than those with normal weight, they have poorer muscle quality. Subsequently, weight loss decreases the skeletal muscle mass without adverse effects on muscle strength and improves the general physical function due to the reduction of fat mass (Cava et al., 2017; Miller and Wolfe, 2008; Gill et al., 2015). Previous research has reported that HU is useful in evaluating muscle degeneration (Ukai et al., 2021). In our study, the HU of the psoas muscle obtained from abdominal CT images showed no significant changes, suggesting that sleeve gastrectomy reduced the psoas muscle mass without changing the muscle quality.

As sleeve gastrectomy affects sex hormones, it may also affect bone metabolism (Yang et al., 2022; Zhu et al., 2019; Emami et al., 2021). As previously reported about the sex-specific difference in bone mass after sleeve gastrectomy, baseline BMD was higher in males than in females, and males had greater weight loss (Yang et al., 2022). Furthermore, males showed a significant decrease in BMD of the total proximal femur and femoral neck as assessed by DXA compared with females after sleeve surgery, although the BMD of the lumbar spine remained unchanged (Risi et al., 2022). The results of the present study are generally consistent with these findings.

The strength of our study was confirming the change in the postoperative bone density of the lumbar spine and psoas muscle by analyzing of HU from simple abdominal CT scans. However, our study had several limitations. First, it was a retrospective study analyzing clinical data. Second, the study duration was short, i.e., analyzing 1 year postoperatively; nevertheless, studies analyzing the long-term duration of bariatric surgery have demonstrated no remarkable changes in the 1-year follow-up, as reported previously (Ben-Porat et al., 2022; Malinici et al., 2021; Vilarrasa et al., 2011). Third, we analyzed only the BMD of the lumbar spine using CT images. A previous study demonstrated that abdominal CT images could identify patients as having osteoporosis or as normal using HU of the spine (Pickhardt et al., 2013). Moreover, analysis using only abdominal CT images could avoid the risk of additional radiation exposure to the proximal femur. Hence, we did not analyze the bone density of the total proximal femur and femoral neck. Yang et al. (2022) demonstrated that BMD measured at 6 months and 1 year after sleeve gastrectomy decreased in the total proximal femur and femoral neck, but not in the lumbar spine. Similarly, our study patients also showed no changes in BMD of the lumbar spine, but it was unclear in the total proximal femur and femoral neck. Fourth, there have been reports on HU variability due to differences in the scanner, scan parameters, and reconstruction protocols (Free et al., 2018). Although no HU calibration was performed in this study, the use of automatic exposure control of the CT allows accurate HU measurements without the use of a phantom (Schreiber et al., 2011). Due to the strong correlation between DXA and HU obtained from clinical abdominal CT scans, BMD assessment using clinical CT scans has been reported to be useful (Schreiber et al., 2011; Lee et al., 2013). Furthermore, scanners in the same facility are used, with matching scan and reconstruction parameters. Fifth, this study did not use a software-based 3D analysis; however, the standardized bone density was examined by measuring the HU of the multiple lumbar spine (L2, L3, L4) of the horizontal sections of clinical abdominal CT images. Sixth, CT scans have disadvantages such as higher radiation exposure, higher medical costs, and limited facilities for imaging. Seventh, although no calcium or vitamin D supplementation was prescribed, there might be some patients purchasing over-the-counter supplements. Finally, although our results demonstrated a significantly decreased psoas muscle mass, we did not assess the muscle strength and functional outcome. Future studies must examine muscle strength through active rehabilitation intervention.

## 5 Conclusions

Sleeve gastrectomy could dramatically improve body weight and body composition without causing changes in serum calcium and phosphorus levels. Preoperative and postoperative abdominal CT revealed that the bone density of the lumbar spine showed no significant differences and that the psoas muscle mass was significantly decreased after sleeve gastrectomy.

## CRediT authorship contribution statement

Study design: KK, TF, and YN. Study conduct and data collection: KK, TF, and YN. Data analysis: KK, TF, and YN. Data interpretation: all authors. Drafting: KK and TF. Revising manuscript content: all authors. Approving final version of manuscript: all authors. TF takes responsibility for the integrity of the data analysis.

## Declaration of competing interest

All authors have no conflict of interests related to this study.

## Data Availability

Data will be made available on request.

## References

[bb0005] Altawil E., Alkofide H., Alamri H., Alhassan N., Alsubaie H., Alqahtani A. (2021). Secondary hyperparathyroidism in obese patients post sleeve gastrectomy. Diabetes Metab. Syndr. Obes..

[bb0010] Ben-Porat T., Peretz S., Rottenstreich A., Weiss R., Szalat A., Elazary R. (2022). Changes in bone mineral density following laparoscopic sleeve gastrectomy: 2-year outcomes. Surg. Obes. Relat. Dis..

[bb0015] Bolotin H.H. (1998). A new perspective on the causal influence of soft tissue composition on DXA-measured in vivo bone mineral density. J. Bone Miner. Res..

[bb0020] Bower J.K., Meadows R.J., Foster M.C., Foraker R.E., Shoben A.B. (2017). The association of percent body fat and lean mass with HbA(1c) in US adults. J. Endocr. Soc..

[bb0025] Bredella M.A., Greenblatt L.B., Eajazi A., Torriani M., Yu E.W. (2017). Effects of roux-en-Y gastric bypass and sleeve gastrectomy on bone mineral density and marrow adipose tissue. Bone.

[bb0030] Cadart O., Degrandi O., Barnetche T., Mehsen-Cetre N., Monsaingeon-Henry M., Pupier E. (2020). Long-term effects of roux-en-Y gastric bypass and sleeve gastrectomy on bone mineral density: a 4-year longitudinal study. Obes. Surg..

[bb0035] Cava E., Yeat N.C., Mittendorfer B. (2017). Preserving healthy muscle during weight loss. Adv. Nutr..

[bb0040] Compston J.E., Bhambhani M., Laskey M.A., Murphy S., Khaw K.T. (1992). Body composition and bone mass in post-menopausal women. Clin. Endocrinol..

[bb0045] Coral R.V., Bigolin A.V., Machry M.C., Menguer R.K., Pereira-Lima J.C., Contin I. (2021). Improvement in muscle strength and metabolic parameters despite muscle mass loss in the initial six months after bariatric surgery. Obes. Surg..

[bb0050] Damms-Machado A., Friedrich A., Kramer K.M., Stingel K., Meile T., Küper M.A. (2012). Pre- and postoperative nutritional deficiencies in obese patients undergoing laparoscopic sleeve gastrectomy. Obes. Surg..

[bb0055] Emami M.R., Safabakhsh M., Khorshidi M., Moradi Moghaddam O., Mohammed S.H., Zarezadeh M. (2021). Effect of bariatric surgery on endogenous sex hormones and sex hormone-binding globulin levels: a systematic review and meta-analysis. Surg. Obes. Relat. Dis..

[bb0060] Evans W.J. (2010). Skeletal muscle loss: cachexia, sarcopenia, and inactivity. Am. J. Clin. Nutr..

[bb0065] Ezzati M. (2016). Trends in adult body-mass index in 200 countries from 1975 to 2014: a pooled analysis of 1698 population-based measurement studies with 19·2 million participants. Lancet.

[bb0070] Free J., Eggermont F., Derikx L., van Leeuwen R., van der Linden Y., Jansen W. (2018). The effect of different CT scanners, scan parameters and scanning setup on hounsfield units and calibrated bone density: a phantom study. Biomed. Phys. Eng. Express.

[bb0075] Gagnon C., Schafer A.L. (2018). Bone health after bariatric surgery. JBMR Plus..

[bb0080] Gill L.E., Bartels S.J., Batsis J.A. (2015). Weight management in older adults. Curr. Obes. Rep..

[bb0085] Griggs C.L., Perez N.P., Goldstone R.N., Kelleher C.M., Chang D.C., Stanford F.C. (2018). National Trends in the use of metabolic and bariatric surgery among pediatric patients with severe obesity. JAMA Pediatr..

[bb0090] Hamaguchi Y., Kaido T., Okumura S., Fujimoto Y., Ogawa K., Mori A. (2014). Impact of quality as well as quantity of skeletal muscle on outcomes after liver transplantation. Liver Transpl..

[bb0095] Hofso D., Hillestad T.O.W., Halvorsen E., Fatima F., Johnson L.K., Lindberg M. (2021). Bone mineral density and turnover after sleeve gastrectomy and gastric bypass: a randomized controlled trial (Oseberg). J. Clin. Endocrinol. Metab..

[bb0100] Jaruvongvanich V., Vantanasiri K., Upala S., Ungprasert P. (2019). Changes in bone mineral density and bone metabolism after sleeve gastrectomy: a systematic review and meta-analysis. Surg. Obes. Relat. Dis..

[bb0105] Kenngott H.G., Nickel F., Wise P.A., Wagner F., Billeter A.T., Nattenmüller J. (2019). Weight loss and changes in adipose tissue and skeletal muscle volume after laparoscopic sleeve gastrectomy and roux-en-Y gastric bypass: a prospective study with 12-month follow-up. Obes. Surg..

[bb0110] Kobayashi A., Kaido T., Hamaguchi Y., Okumura S., Taura K., Hatano E. (2016). Impact of postoperative changes in sarcopenic factors on outcomes after hepatectomy for hepatocellular carcinoma. J. Hepatobiliary Pancreat. Sci..

[bb0115] Lancha A., Moncada R., Valentí V., Rodríguez A., Catalán V., Becerril S. (2014). Comparative effects of gastric bypass and sleeve gastrectomy on plasma osteopontin concentrations in humans. Surg. Endosc..

[bb0120] Lee S., Chung C.K., Oh S.H., Park S.B. (2013). Correlation between bone mineral density measured by dual-energy X-ray absorptiometry and hounsfield units measured by diagnostic CT in lumbar spine. J. Korean Neurosurg. Soc..

[bb0125] Levitt D.G., Beckman L.M., Mager J.R., Valentine B., Sibley S.D., Beckman T.R. (2010). Comparison of DXA and water measurements of body fat following gastric bypass surgery and a physiological model of body water, fat, and muscle composition. J. Appl. Physiol. (1985).

[bb0130] Lindsay R., Cosman F., Herrington B.S., Himmelstein S. (1992). Bone mass and body composition in normal women. J. Bone Miner. Res..

[bb0135] Liu C., Wu D., Zhang J.F., Xu D., Xu W.F., Chen Y. (2016). Changes in bone metabolism in morbidly obese patients after bariatric surgery: a meta-analysis. Obes. Surg..

[bb0140] Lucas K., Behrens B.A., Nolte I., Galindo-Zamora V., Betancur S., Almohallami A. (2017). Comparative investigation of bone mineral density using CT and DEXA in a canine femoral model. J. Orthop. Res..

[bb0145] Malinici E., Sirbu A., Popa M., Andrei M., Ioacara S., Copaescu C. (2021). Bone mineral density trends during the first year after laparoscopic sleeve gastrectomy-a cohort study on 241 patients. Obes. Surg..

[bb0150] Marshall D., Johnell O., Wedel H. (1996). Meta-analysis of how well measures of bone mineral density predict occurrence of osteoporotic fractures. BMJ.

[bb0155] Mechanick J.I., Kushner R.F., Sugerman H.J., Gonzalez-Campoy J.M., Collazo-Clavell M.L., Spitz A.F. (2009). American Association of Clinical Endocrinologists, The Obesity Society, and American Society for Metabolic & Bariatric Surgery medical guidelines for clinical practice for the perioperative nutritional, metabolic, and nonsurgical support of the bariatric surgery patient. Obesity (Silver Spring).

[bb0160] Miller S.L., Wolfe R.R. (2008). The danger of weight loss in the elderly. J. Nutr. Health Aging.

[bb0165] Misra M., Singhal V., Carmine B., Bose A., Kelsey M.M., Stanford F.C. (2020). Bone outcomes following sleeve gastrectomy in adolescents and young adults with obesity versus non-surgical controls. Bone.

[bb0170] Moize V., Andreu A., Rodriguez L., Flores L., Ibarzabal A., Lacy A. (2013). Protein intake and lean tissue mass retention following bariatric surgery. Clin. Nutr..

[bb0175] Molero J., Olbeyra R., Flores L., Jiménez A., de Hollanda A., Andreu A. (2022). Prevalence of low skeletal muscle mass following bariatric surgery. Clin. Nutr. ESPEN.

[bb0180] Otto M., Kautt S., Kremer M., Kienle P., Post S., Hasenberg T. (2014). Handgrip strength as a predictor for post bariatric body composition. Obes. Surg..

[bb0185] Ozeki Y., Masaki T., Yoshida Y., Okamoto M., Anai M., Gotoh K. (2018). Bioelectrical impedance analysis results for estimating body composition are associated with glucose metabolism following laparoscopic sleeve gastrectomy in obese japanese patients. Nutrients.

[bb0190] Peterli R., Wölnerhanssen B.K., Peters T., Vetter D., Kröll D., Borbély Y. (2018). Effect of laparoscopic sleeve gastrectomy vs laparoscopic roux-en-Y gastric bypass on weight loss in patients with morbid obesity: the SM-BOSS randomized clinical trial. JAMA.

[bb0195] Pickhardt P.J., Pooler B.D., Lauder T., del Rio A.M., Bruce R.J., Binkley N. (2013). Opportunistic screening for osteoporosis using abdominal computed tomography scans obtained for other indications. Ann. Intern. Med..

[bb0200] Risi R., Rossini G., Tozzi R., Pieralice S., Monte L., Masi D. (2022). Sex difference in the safety and efficacy of bariatric procedures: a systematic review and meta-analysis. Surg. Obes. Relat. Dis..

[bb0205] Ruiz-Tovar J., Oller I., Priego P., Arroyo A., Calero A., Diez M. (2013). Short- and mid-term changes in bone mineral density after laparoscopic sleeve gastrectomy. Obes. Surg..

[bb0210] Santos D., Lopes T., Jesus P., Cruz S., Cordeiro A., Pereira S. (2019). Bone metabolism in adolescents and adults undergoing roux-en-Y gastric bypass: a comparative study. Obes. Surg..

[bb0215] Schauer P.R., Bhatt D.L., Kirwan J.P., Wolski K., Brethauer S.A., Navaneethan S.D. (2014). Bariatric surgery versus intensive medical therapy for diabetes–3-year outcomes. N. Engl. J. Med..

[bb0220] Schreiber J.J., Anderson P.A., Rosas H.G., Buchholz A.L., Au A.G. (2011). Hounsfield units for assessing bone mineral density and strength: a tool for osteoporosis management. J. Bone Joint Surg. Am..

[bb0225] Slemenda C.W., Hui S.L., Williams C.J., Christian J.C., Meaney F.J., Johnston C.C. (1990). Bone mass and anthropometric measurements in adult females. Bone Miner..

[bb0230] Stegen S., Derave W., Calders P., Van Laethem C., Pattyn P. (2011). Physical fitness in morbidly obese patients: effect of gastric bypass surgery and exercise training. Obes. Surg..

[bb0235] Switzer N.J., Prasad S., Debru E., Church N., Mitchell P., Gill R.S. (2016). Sleeve gastrectomy and type 2 diabetes mellitus: a systematic review of long-term outcomes. Obes. Surg..

[bb0240] Tothill P., Hannan W.J., Cowen S., Freeman C.P. (1997). Anomalies in the measurement of changes in total-body bone mineral by dual-energy X-ray absorptiometry during weight change. J. Bone Miner. Res..

[bb0245] Ukai T., Ebihara G., Omura H., Watanabe M. (2021). Evaluation of muscle volume and degeneration after total hip arthroplasty: a comparison of the posterolateral approach and the anterolateral supine approach. J. Orthop. Surg. Res..

[bb0250] Vaurs C., Dimeglio C., Charras L., Anduze Y., Chalret du Rieu M., Ritz P. (2015). Determinants of changes in muscle mass after bariatric surgery. Diabetes Metab..

[bb0255] Vilarrasa N., San José P., García I., Gómez-Vaquero C., Miras P.M., de Gordejuela A.G. (2011). Evaluation of bone mineral density loss in morbidly obese women after gastric bypass: 3-year follow-up. Obes. Surg..

[bb0260] Yang D., Ye Y., Tu Y., Xu R., Xiao Y., Zhang H. (2022). Sex-specific differences in bone mineral density loss after sleeve gastrectomy. Front. Med. (Lausanne).

[bb0265] Yu E.W., Thomas B.J., Brown J.K., Finkelstein J.S. (2012). Simulated increases in body fat and errors in bone mineral density measurements by DXA and QCT. J. Bone Miner. Res..

[bb0270] Zhu C., Zhang Y., Zhang L., Gao J., Mei F., Zhu B. (2019). Changes in sex hormones after laparoscopic sleeve gastrectomy in chinese obese men: a 12-month follow-up. Obes. Surg..

